# Association Between the Implementation of Shared Decision-Making and Clinical Characteristics of Living-Donor Kidney Transplantation

**DOI:** 10.3390/healthcare14172774

**Published:** 2026-09-01

**Authors:** Akito Mochizuki, Kazuhiro Tada, Sho Shimamoto, Koji Takahashi, Kenji Ito, Yoko Yokoyama, Masahiro Tachibana, Nobuyuki Nakamura, Nobuhiro Haga, Kosuke Masutani

**Affiliations:** 1 Division of Nephrology and Rheumatology, Department of Internal Medicine, Faculty of Medicine, Fukuoka University, Fukuoka 814-0180, Japan; 2 Department of Nursing, Fukuoka University Hospital, Fukuoka 814-0180, Japan; 3 Department of Urology, Faculty of Medicine, Fukuoka University, Fukuoka 814-0180, Japan

**Keywords:** ABO-incompatible kidney transplantation, chronic kidney disease, outpatient clinic, patient-centered care, preemptive kidney transplantation

## Abstract

**Background/Objectives:** Shared decision-making (SDM), which incorporates patients’ values and preferences, may support patient-centered selection of renal replacement therapy, but its association with the clinical characteristics of living-donor kidney transplantation (LDKT) remains unclear. This study aimed to evaluate the association between SDM implementation and the clinical characteristics of LDKT. **Methods:** A specialized SDM outpatient clinic was established at our institution in 2019. We retrospectively analyzed 65 patients who underwent LDKT at a single center between 2010 and 2025, comparing a pre-SDM group (2010–2018, *n* = 25) with a post-SDM group (2019–2025, *n* = 40). **Results:** Recipient characteristics were similar between groups, whereas donors were older after SDM implementation (62.0 [52.3–68.0] vs. 55.0 [44.5–60.0] years; *p* = 0.01). ABO-incompatible transplantation (37.5% vs. 4.0%; *p* = 0.003) and preformed donor-specific antibody-positive transplantation (20.0% vs. 0%; *p* = 0.02) were more frequent in the post-SDM group. The proportion of preemptive kidney transplantation (PEKT) was similar; however, among PEKT recipients, estimated glomerular filtration rate was higher at initial evaluation and admission, and preoperative dialysis was numerically less frequent in the post-SDM group (20.0% vs. 54.5%; *p* = 0.106). **Conclusions:** Implementation of the SDM clinic was associated with a higher frequency of immunologically complex LDKT and better-preserved kidney function at initial transplant evaluation and admission among PEKT recipients. These associations should be interpreted cautiously given potential secular changes in transplant practice.

## 1. Introduction

Among renal replacement therapy (RRT) modalities for patients with end-stage kidney disease, hemodialysis, peritoneal dialysis, and kidney transplantation are available. Of these, kidney transplantation is the most effective treatment, with clear advantages in survival and quality of life [1,2]. However, while deceased-donor kidney transplantation predominates in some countries, a shortage of deceased donors has led to dependence on living-donor kidney transplantation (LDKT) in Japan and many other countries [3]. To expand the indications for living donation, extensive basic and clinical research on ABO-incompatible kidney transplantation (ABOi-KT) has been conducted in Japan, with outcomes comparable to those of ABO-compatible transplantation [4]. Additionally, preemptive kidney transplantation (PEKT) has been increasingly performed in recent years. PEKT has been associated with improved graft survival and reduced cardiovascular events, with growing evidence from retrospective studies and meta-analyses [5].

Recently, patient-centered care has emphasized the importance of shared decision-making (SDM), a process in which patients and clinicians collaboratively determine treatment options [6]. In the context of RRT, SDM has been shown to improve informed, patient-centered decision-making and patient satisfaction [7], with reports of increased selection of peritoneal dialysis and a higher proportion of patients undergoing pre-transplant evaluation following its introduction [8]. By providing patients and families with comprehensive information about the risks and benefits of LDKT, SDM may facilitate consideration of transplantation involving older donors or immunologically complex conditions, such as ABO incompatibility and preformed donor-specific anti-HLA antibody (DSA) positivity. In addition, earlier discussion of transplantation as an RRT option may prompt earlier donor and recipient evaluation, potentially allowing PEKT to be planned while kidney function is better preserved. However, although some studies have described changes in transplant selection following SDM implementation, differences in the clinical characteristics of transplantation associated with its implementation remain unclear. Therefore, this study aimed to examine differences in the clinical characteristics of LDKT before and after implementation of a structured SDM outpatient clinic.

## 2. Materials and Methods

### 2.1. Shared Decision-Making Outpatient Clinic

A specialized SDM outpatient clinic was established at Fukuoka University Hospital in April 2019. Following routine chronic kidney disease (CKD) outpatient visits, patients were allocated approximately 60 min per session across two to three sessions, during which a dedicated nurse conducted structured information gathering and provided explanations about treatment options. The nurse elicited each patient’s work and lifestyle, family circumstances, ability to attend regular medical visits, and preferences and concerns regarding RRT. In addition to written educational materials, patients and their families were offered hands-on exposure to peritoneal dialysis connection devices, automated peritoneal dialysis machines, educational DVDs, and tours of the hemodialysis unit. Treatment options were explained in the following order: kidney transplantation, peritoneal dialysis, and hemodialysis. Patients aged ≥75 years, those with untreatable malignancies, or those with severe cardiovascular comorbidities were considered unsuitable candidates for kidney transplantation. SDM was generally initiated in patients with CKD stage G4 or G5. Although no strict eGFR threshold was used, the timing of referral was guided by the expected time to end-stage kidney disease, taking into account the rate of kidney function decline. In principle, patients expected to require kidney replacement therapy within approximately one year were referred to the SDM clinic. For older patients, earlier referral, sometimes 2–3 years in advance, was considered because cognitive function, physical function, and overall clinical status could change substantially over time. These general referral principles remained consistent throughout the post-SDM period, although the final timing of referral was determined by the attending nephrologist’s clinical judgment. After information on the available RRT modalities was provided, the advantages and disadvantages of each option were discussed with the patient and family in the context of the patient’s individual circumstances and preferences. Patient understanding was confirmed verbally during these discussions. The final RRT modality was selected through discussion among the patient, family members, nurses, and physicians and was documented in the electronic medical record. Institution-specific educational materials and checklists were used to support and standardize this process; however, no validated decision aid or formal SDM framework was used.

### 2.2. Study Design and Participants

This retrospective cohort study included 65 patients who underwent LDKT between 2010 and 2025. Patients who received LDKT prior to the introduction of SDM were classified as the pre-SDM group (2010–2018, *n* = 25), and those thereafter were defined as the post-SDM group (2019–2025, *n* = 40). Donor and recipient demographic and clinical characteristics, transplant-related factors (ABOi-KT, DSA positivity, and PEKT), and preoperative kidney function (serum creatinine concentration and eGFR) were compared. eGFR was calculated using the equation developed for the Japanese population [9]. Hypertension was defined as a blood pressure of ≥140/90 mmHg at the outpatient clinic or treatment with antihypertensive drugs. Diabetes mellitus was defined as a fasting plasma glucose of ≥126 mg/dL, HbA1c of ≥6.5%, 2 h plasma glucose of ≥200 mg/dL during a 75 g oral glucose tolerance test, or current use of hypoglycemic therapy. PEKT was defined as kidney transplantation performed prior to the initiation of maintenance dialysis; patients requiring temporary hemodialysis via a catheter for conditioning purposes were also included. In patients undergoing PEKT, kidney function at the initiation of evaluation and at admission for transplantation, as well as the need for preoperative hemodialysis, were assessed.

### 2.3. Statistical Analysis

Statistical analyses were performed using JMP Student Edition 18.2.1 (SAS Institute, Cary, NC, USA). Continuous variables were expressed as medians with interquartile ranges, and categorical variables were expressed as counts with percentages. Comparisons between the two groups were conducted using the Mann–Whitney U test for continuous variables. Categorical variables were compared using the chi-square test or Fisher’s exact test, with Fisher’s exact test used when any expected cell count was <5. For categorical variables with more than two levels, an overall test comparing the distribution of categories between the pre-SDM and post-SDM groups was performed. All analyses were exploratory, and no adjustment for multiple comparisons was performed. A two-tailed *p* value of <0.05 was considered statistically significant. For principal continuous outcomes, Hodges–Lehmann estimates of location shift with 95% confidence intervals were calculated. For principal binary outcomes, risk differences with 95% confidence intervals were calculated using the adjusted Wald method. The study protocol was approved by the Ethics Committee of Fukuoka University Hospital (IRB H21-08-004), and all transplantations were performed in accordance with the Declaration of Istanbul. All living donors were either biologically related to the recipients (within the fourth degree of consanguinity) or were spouses, and were aged 18 years or older. No living unrelated donors were included in this study.

## 3. Results

Between 2019 and 2025, a total of 412 patients with stage G4/G5 CKD attended the SDM clinic. Of these, 247 (60.0%) initially selected HD, 109 (26.5%) selected PD, 29 (7.0%) selected LDKT, and 27 (6.6%) selected conservative kidney management. Initial treatment choices did not necessarily correspond to the modalities ultimately received. Some patients who initially selected LDKT subsequently became medically ineligible for transplantation and received dialysis instead, whereas some who initially selected HD or PD subsequently pursued LDKT and underwent transplantation after dialysis initiation. Therefore, the 29 patients who initially selected LDKT did not directly correspond to the 40 LDKT recipients in the post-SDM cohort. Among these 40 recipients, 34 (85.0%) had attended the SDM clinic, including all 20 PEKT recipients and 14 of the 20 non-PEKT recipients. The remaining six patients were referred directly from other institutions for LDKT and had not attended our SDM clinic.

Demographic and clinical characteristics of recipients and donors, as well as transplant-related factors, are summarized in Table 1, Table 2 and Table 3, respectively. Recipient age, sex, and primary kidney disease did not differ significantly between groups (Table 1). Donor age, however, was significantly higher in the post-SDM group than in the pre-SDM group (62.0 [52.3–68.0] vs. 55.0 [44.5–60.0] years, Hodges–Lehmann location shift, 7.0 years; 95% CI, 1.0–12.0; *p* = 0.01). No significant differences were observed between groups in donor hypertension, diabetes, or baseline kidney function (Table 2). The specific degree of relationship between donors and recipients is also summarized in Table 2.

With regard to transplant-related factors, the proportion of ABOi-KT was significantly higher in the post-SDM group (37.5% vs. 4.0%; risk difference, 33.5 percentage points; 95% CI, 13.0–48.8; *p* = 0.003). No DSA-positive transplantations were performed in the pre-SDM group, whereas they were performed in the post-SDM group (20.0%), showing a significant difference (risk difference, 20.0 percentage points; 95% CI, 3.4–32.0; *p* = 0.02) (Table 3).

Clinical characteristics of the patients who underwent PEKT are summarized separately in Table 4. The proportion of PEKT was comparable between the pre- and post-SDM groups (44.0% and 50.0%, respectively). However, initial eGFR at evaluation was significantly higher in the post-SDM group (11.4 [9.4–13.7] vs. 7.7 [7.1–9.5] mL/min/1.73 m^2^, Hodges–Lehmann location shift, 3.5 mL/min/1.73 m^2^; 95% CI, 1.1–5.9; *p* = 0.01). The time from evaluation to transplantation was 118.0 [101.0–188.5] days in the pre-SDM group and 179.0 [119.0–243.0] days in the post-SDM group, without a significant difference. At admission for transplantation, eGFR was higher in the post-SDM group (8.7 [6.8–10.6] vs. 5.8 [4.9–7.3] mL/min/1.73 m^2^, Hodges–Lehmann location shift, 2.3 mL/min/1.73 m^2^; 95% CI, 0.6–4.5; *p* = 0.006). Pre-transplant hemodialysis via a temporary catheter was less frequent in the post-SDM group than in the pre-SDM group, although the difference did not reach statistical significance (20.0% vs. 54.5%; risk difference, −34.5 percentage points; 95% CI, −63.4–1.1; *p* = 0.106).

## 4. Discussion

This study examined differences in the clinical characteristics of LDKT before and after the introduction of SDM in RRT selection. We found that the introduction of SDM was associated with older donor age and higher frequencies of ABOi-KT and preformed DSA-positive kidney transplantation. Furthermore, among PEKT recipients, eGFR at initial transplant evaluation and admission was higher in the post-SDM group. To our knowledge, this is the first report describing an association between the implementation of SDM and the clinical characteristics of subsequent LDKT.

In Japan, the age at dialysis initiation has been steadily increasing [10], paralleled by increasing recipient age in transplantation [11]. In our study, although recipient age did not differ significantly, a slight upward trend was observed. Donor age was significantly higher in the post-SDM group than in the pre-SDM group. This difference may reflect multiple factors, including secular changes in transplant practice and increasing experience with older living donors. Although improved information sharing and deliberation through SDM may have contributed to the consideration of older donors by patients and families, this remains a hypothesis that cannot be established by the present study. Previous studies have shown that patients’ decisions to choose or decline kidney transplantation are influenced by multiple factors, including the information provided [12].

Furthermore, in Japan, limited understanding of ABOi-KT may also exist. These misconceptions may be addressed through appropriate information provision and sufficient time for SDM, which could ultimately improve access to kidney transplantation and expand its indications. Importantly, there was no major change in institutional eligibility criteria or the fundamental desensitization strategy coinciding with the introduction of SDM in 2019. Rituximab and double-filtration plasmapheresis had already been used for ABOi transplantation at our institution before the introduction of SDM. Therefore, although broader secular changes in transplant practice cannot be excluded, the increase in ABOi-KT after 2019 was unlikely to be explained solely by a contemporaneous procedural change at our institution. In contrast, the increase in preformed DSA-positive transplantation should be interpreted more cautiously. Complement-dependent cytotoxicity crossmatch, flow cytometric crossmatch, and Luminex bead-based assays had been established from the beginning of the study period, and the overall DSA assessment strategy, positivity threshold, and approach to immunological risk stratification remained essentially unchanged, although minor changes in Luminex assay products occurred during the study period. In contrast, advances in desensitization therapy, including the approval of intravenous immunoglobulin for DSA-positive kidney transplantation in 2019 and rituximab in 2023, increased the clinical feasibility and acceptance of DSA-positive transplantation and may have contributed to its higher frequency in the post-SDM period.

Among PEKT recipients, eGFR at both initial transplant evaluation and admission was significantly higher in the post-SDM group, indicating better-preserved kidney function at these time points. Although this may be consistent with earlier consideration of transplantation, the present study cannot establish that SDM itself resulted in earlier evaluation. Preoperative hemodialysis was numerically less frequent in the post-SDM group (20.0% vs. 54.5%), but the difference was not statistically significant (*p* = 0.106). Kidney transplant recipients are typically patients with CKD stage G5, and evaluation of surgical tolerance is crucial, particularly screening for cardiovascular disease. Because postoperative immunosuppressive therapy is required, screening for malignancy and infections is also performed, aiming for curative treatment if detected, and vaccinations are administered when necessary. For donors, overall kidney function, cardiopulmonary and hepatic function, and the presence of infections or malignancies are assessed, along with psychological counseling to ensure long-term physical and mental safety after donation. If any problems are identified during this process, donor substitution is considered; indeed, in this study, donor changes were made in seven cases. The necessity of preoperative hemodialysis is determined individually, without clear criteria; however, it was performed when perioperative complications were anticipated, such as bleeding tendency due to uremia, fluid overload, or hyperkalemia. Although the difference in preoperative hemodialysis did not reach statistical significance, the lower numerical frequency observed in the post-SDM group may reflect more planned preparation for PEKT. Furthermore, a more planned transition to transplantation may have potential clinical and health-service benefits by avoiding emergency vascular access and unnecessary hospitalization [13], although these outcomes were not evaluated in the present study.

In the selection of RRT, SDM is recognized as an effective approach that supports treatment choices aligned with patients’ values and lifestyles. Although patients with CKD face multiple treatment options, including hemodialysis, peritoneal dialysis, kidney transplantation, and conservative kidney management, SDM encourages collaborative dialog between patients and healthcare providers, leading to improved satisfaction and adherence. Elwyn et al. [6] described SDM as a framework for patient-centered care and quality improvement in healthcare. Morton et al. [14] highlighted that decision-making in CKD is complex and often associated with anxiety and confusion, suggesting that improved decision support and SDM could be beneficial. Importantly, SDM has also been shown to promote selection of treatment modalities that have historically been underutilized, such as peritoneal dialysis and kidney transplantation. Similar findings have been reported in other countries. In Taiwan, Lee et al. reported that implementation of an SDM program for RRT was associated with increased selection of peritoneal dialysis and a higher proportion of patients undergoing pre-transplant evaluation [8]. Similarly, in the United Kingdom, Winterbottom et al. reported that incorporating a patient decision aid into pre-dialysis education supported patients’ understanding of kidney disease, consideration of treatment options, and involvement of family members in decision making [15]. While previous international studies have primarily focused on the decision-making process and RRT modality selection, the present study focused on the clinical characteristics of patients who ultimately underwent LDKT. Our findings complement these studies by describing differences in the characteristics and timing of LDKT observed after implementation of an SDM clinic.

This study has several limitations. First, it was a single-center retrospective study with a small sample size, limiting statistical power and generalizability. Because multiple exploratory comparisons were performed without adjustment for multiplicity, the possibility of type I error should be considered. In addition, the small sample size and sparse events precluded reliable multivariable adjustment for potential confounders. Second, the pre-SDM and post-SDM groups were defined by calendar period rather than individual exposure to the SDM intervention. Although 34 of the 40 patients (85.0%) in the post-SDM group attended the SDM clinic, six patients referred directly for LDKT did not. Consequently, secular changes in transplant practice—including increasing experience with ABOi-KT, DSA-positive transplantation, PEKT, and older living donors—may have contributed to the observed differences independently of SDM. The study design therefore cannot establish a causal effect of SDM. Third, selection bias may have occurred because the analytical cohort was restricted to patients who ultimately underwent LDKT. Because a comparable denominator of patients undergoing RRT decision-making was not available for the pre-SDM period, this study cannot determine whether implementation of the SDM clinic was associated with an increased likelihood of selecting or receiving LDKT relative to other modalities. Fourth, preoperative dialysis decisions were physician-dependent, potentially introducing treatment-selection bias. Fifth, unmeasured confounders such as socioeconomic status, education, and health literacy may have influenced outcomes. Future large-scale, prospective, and ideally randomized controlled studies are needed to confirm our findings. An ongoing randomized trial in Korea comparing SDM with standard care may provide further insights [16]. Finally, SDM participation and intervention fidelity were not assessed using validated measures; therefore, the degree of individual patient engagement and the consistency of intervention delivery could not be quantified.

## 5. Conclusions

The introduction of a structured SDM outpatient clinic for RRT selection was associated with changes in the clinical characteristics and timing of LDKT at our center. After SDM implementation, transplantation involving older donors, ABO incompatibility, and preformed DSA positivity was more frequent. Among PEKT recipients, kidney function was better preserved at initial transplant evaluation and at admission in the post-SDM group. Because this historical pre–post study cannot separate the effect of SDM from secular changes in transplant practice, these findings should be interpreted as associations. Prospective studies are warranted to further evaluate the role of structured SDM in RRT decision-making and kidney transplantation.

## Figures and Tables

**Table 1:** Demographic and clinical characteristics of the kidney transplant recipients.

	Overall (*n* = 65)	Pre-SDM Group (*n* = 25)	Post-SDM Group (*n* = 40)	*p* Value
Age at transplantation (years)	39.0 [32.0–51.0]	37.0 [27.0–45.0]	41.5 [35.0–51.3]	0.08
Male sex	44 (67.7)	20 (80.0)	24 (60.0)	0.09
BMI at transplantation, kg/m^2^	21.2 [19.9–23.7]	21.0 [19.9–23.7]	21.4 [19.7–23.8]	0.90
Primary kidney disease				
Chronic glomerulonephritis	17 (26.2)	5 (20.0)	12 (30.0)	0.65
Diabetic nephropathy	15 (23.1)	5 (20.0)	10 (25.0)	
Reflux nephropathy	6 (9.2)	3 (12.0)	3 (7.5)	
Renal hypoplasia	6 (9.2)	3 (12.0)	3 (7.5)	
Hypertensive nephrosclerosis	5 (7.7)	2 (8.0)	3 (7.5)	
Chronic tubulointerstitial nephritis	5 (7.7)	1 (4.0)	4 (10.0)	
Other	11 (16.9)	6 (24.0)	5 (12.5)	
Re-transplantation	4 (6.2)	0 (0.0)	4 (10.0)	0.15
Dialysis duration, months *	9.0 [6.3–30.8]	25.0 [5.5–38.3]	9.0 [6.8–16.3]	0.21
Maintenance dialysis	34 (52.3)	14 (56.0)	20 (50.0)	0.64
Dialysis modality				
Hemodialysis	28 (43.1)	13 (52.0)	15 (37.5)	0.36
Peritoneal dialysis	6 (9.2)	1 (4.0)	5 (12.5)	

Data are expressed as median [interquartile range] or n (%). Percentages were calculated based on the total number of patients in each group. Categorical variables were compared using the chi-square test or Fisher’s exact test, as appropriate; Fisher’s exact test was used when any expected cell count was <5. For variables with more than two categories, overall *p* values are reported. * Dialysis duration was calculated only for patients who underwent maintenance dialysis. Abbreviations: BMI, body mass index; SDM, shared decision-making.

**Table 2:** Demographic and clinical characteristics of the living kidney donors.

	Overall (*n* = 65)	Pre-SDM Group (*n* = 25)	Post-SDM Group (*n* = 40)	*p* Value
Age at donation, years	60.0 [51.5–65.3]	55.0 [44.5–60.0]	62.0 [52.3–68.0]	0.01
Male sex	29 (44.6)	10 (40.0)	19 (47.5)	0.55
BMI, kg/m^2^	23.5 [21.5–25.1]	22.0 [21.0–24.3]	23.7 [22.0–25.4]	0.18
Relationship to the recipient				
Parent	38 (58.5)	14 (56.0)	24 (60.0)	0.87
Spouse	17 (26.2)	8 (32.0)	9 (22.5)	
Sibling	8 (12.3)	3 (12.0)	5 (12.5)	
Child	1 (1.5)	0 (0.0)	1 (2.5)	
Uncle	1 (1.5)	0 (0.0)	1 (2.5)	
Hypertension	17 (26.2)	7 (28.0)	10 (25.0)	0.79
Diabetes mellitus	4 (6.2)	0 (0.0)	4 (10.0)	0.15
eGFR at donation, mL/min/1.73 m^2^	76.0 [64.1–84.4]	77.2 [64.1–85.2]	75.9 [64.1–81.2]	0.59
DTPA-GFR at donation, mL/min	85.3 [71.0–98.0]	86.1 [78.2–93.9]	83.8 [70.0–99.0]	0.80

Data are expressed as median [interquartile range] or n (%). Percentages were calculated based on the total number of patients in each group. Categorical variables were compared using the chi-square test or Fisher’s exact test, as appropriate; Fisher’s exact test was used when any expected cell count was <5. For variables with more than two categories, overall *p* values are reported. Abbreviations: BMI, body mass index; SDM, shared decision-making; eGFR, estimated glomerular filtration rate; DTPA, diethylenetriaminepentaacetic acid.

**Table 3:** Summary of transplant-related factors.

	Overall (*n* = 65)	Pre-SDM Group (*n* = 25)	Post-SDM Group (*n* = 40)	*p* Value
ABOi-KT	16 (24.6)	1 (4.0)	15 (37.5)	0.003
Preformed DSA positivity	8 (12.3)	0 (0.0)	8 (20.0)	0.02
HLA-A, -B, -DR mismatch count	3.0 [2.0–3.0]	3.0 [2.0–3.0]	3.0 [2.0–3.0]	0.85
PEKT	31 (47.7)	11 (44.0)	20 (50.0)	0.64

Data are expressed as median [interquartile range] or n (%). Percentages were calculated based on the total number of patients in each group. Categorical variables were compared using the chi-square test or Fisher’s exact test, as appropriate; Fisher’s exact test was used when any expected cell count was <5. Abbreviations: SDM, shared decision-making; ABOi-KT, ABO-incompatible kidney transplantation; DSA, donor-specific antibody; HLA, human leukocyte antigen; PEKT, preemptive kidney transplantation.

**Table 4:** Details of patients who underwent preemptive kidney transplantation.

Variable	Overall (*n* = 31)	Pre-SDM Group (*n* = 11)	Post-SDM Group (*n* = 20)	*p* Value
Serum creatinine at first evaluation, mg/dL	5.4 [4.5–6.7]	7.4 [6.1–7.6]	4.8 [3.9–5.7]	0.001
eGFR at first evaluation, mL/min/1.73 m^2^	9.8 [7.7–12.1]	7.7 [7.1–9.5]	11.4 [9.4–13.7]	0.01
Time from first evaluation to transplantation, days	163.0 [108.3–234.0]	118.0 [101.0–188.5]	179.0 [119.0–243.0]	0.26
Serum creatinine at admission, mg/dL	6.9 [6.0–9.1]	9.3 [8.2–10.7]	6.2 [5.4–7.1]	0.0004
eGFR at admission, mL/min/1.73 m^2^	7.0 [5.7–8.9]	5.8 [4.9–7.3]	8.7 [6.8–10.6]	0.006
Preoperative dialysis	10 (32.3)	6 (54.5)	4 (20.0)	0.106

Data are expressed as median [interquartile range] or n (%). Percentages were calculated based on the total number of patients in each group. Categorical variables were compared using the chi-square test or Fisher’s exact test, as appropriate; Fisher’s exact test was used when any expected cell count was <5. Abbreviations: SDM, shared decision-making; eGFR, estimated glomerular filtration rate.

## Data Availability

The data presented in this study are available from the corresponding author upon reasonable request, subject to institutional ethical and privacy restrictions.
